# The Metabolic Basis of Pollen Thermo-Tolerance: Perspectives for Breeding

**DOI:** 10.3390/metabo4040889

**Published:** 2014-09-30

**Authors:** Marine J. Paupière, Adriaan W. van Heusden, Arnaud G. Bovy

**Affiliations:** 1 Plant Breeding, Wageningen University, Droevendaalsesteeg 1, Wageningen 6708PB, The Netherlands; E-Mail: marine.paupiere@wur.nl; 2 Plant Research International, Wageningen University Plant Breeding, Droevendaalsesteeg 1, Wageningen 6708PB, The Netherlands; E-Mail: sjaak.vanheusden@wur.nl

**Keywords:** pollen, heat stress, thermotolerance, high temperature, metabolite, breeding

## Abstract

Crop production is highly sensitive to elevated temperatures. A rise of a few degrees above the optimum growing temperature can lead to a dramatic yield loss. A predicted increase of 1–3 degrees in the twenty first century urges breeders to develop thermo-tolerant crops which are tolerant to high temperatures. Breeding for thermo-tolerance is a challenge due to the low heritability of this trait. A better understanding of heat stress tolerance and the development of reliable methods to phenotype thermo-tolerance are key factors for a successful breeding approach. Plant reproduction is the most temperature-sensitive process in the plant life cycle. More precisely, pollen quality is strongly affected by heat stress conditions. High temperature leads to a decrease of pollen viability which is directly correlated with a loss of fruit production. The reduction in pollen viability is associated with changes in the level and composition of several (groups of) metabolites, which play an important role in pollen development, for example by contributing to pollen nutrition or by providing protection to environmental stresses. This review aims to underline the importance of maintaining metabolite homeostasis during pollen development, in order to produce mature and fertile pollen under high temperature. The review will give an overview of the current state of the art on the role of various pollen metabolites in pollen homeostasis and thermo-tolerance. Their possible use as metabolic markers to assist breeding programs for plant thermo-tolerance will be discussed.

## 1. Introduction

Environmental stresses are important factors affecting worldwide crop production [1]. Among them, high temperature plays a crucial role and this review will focus on this specific abiotic stress [2]. Heat stress is a complex trait which depends on the duration, the fluctuation and the intensity of temperature rise above the optimal growth temperature [3]. High temperature is one of the major factors limiting the growth season in many parts of Asia, one of the most important producers of daily consumed crops such as rice (*Oryza sativa*), wheat (*Triticum aestivum*), potatoes (*Solanum tuberosum*) and tomatoes (*Solanum lycopersicum*). The expected global warming, with a predicted increase of 1–3 °C during the twenty first century, could therefore have a major impact on agriculture and may lead to significant decreases in crop production [4]. Breeding for thermo-tolerant genotypes is of major importance to maintain crop production under hot conditions. This requires a thorough understanding of the mechanisms underlying heat stress tolerance. 

**Figure 1:**
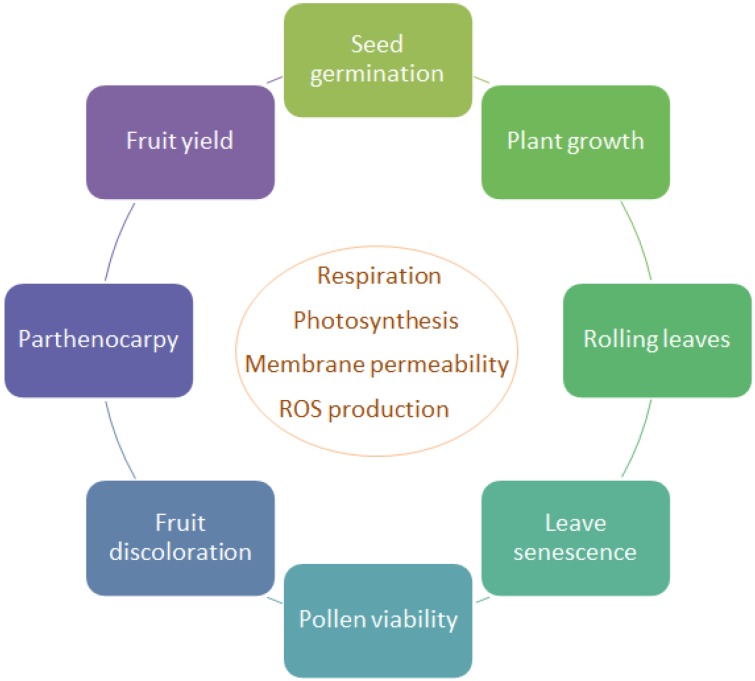
General effects of heat stress on plant physiology.

Plants can respond to heat stress through different mechanisms. High temperature alters plant growth, including rolling of leaves, leaf senescence, root and shoot growth inhibition as well as seed germination reduction, fruit discoloration, decrease in pollen viability and decrease in yield [5], as a result of severe alterations in basic physiological processes, such as increased respiration, decreased photosynthesis, increased membrane permeability and ROS production (Figure 1). A genotype is considered thermo-tolerant when it can produce economic yield under heat stress. Several experimental parameters can be measured to monitor thermo-tolerance [3], including cell membrane thermostability, photosynthesis activity, pollen viability and fruit set. These parameters will be discussed below. 

**Figure 2:**
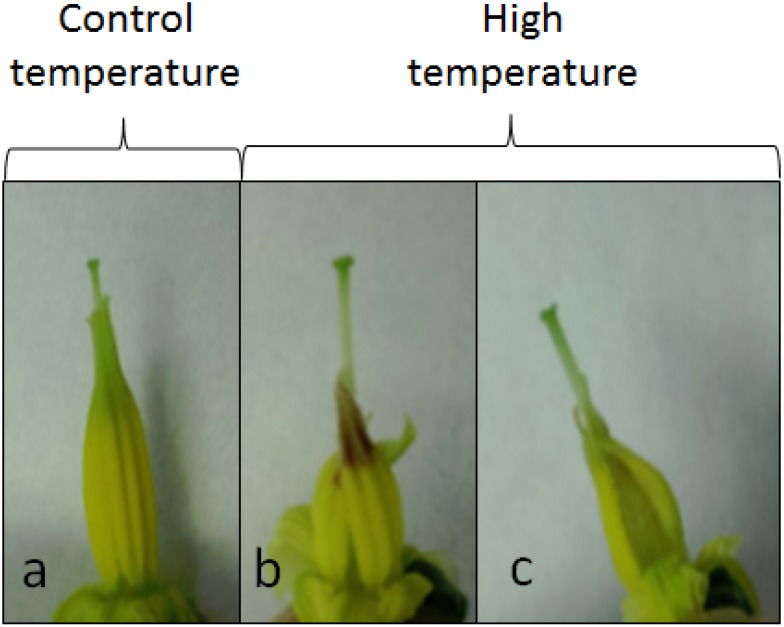
Effect of high temperature (34 °C/28 °C) on flowers of *Solanum lycopersicum* cv. Nagcarlang. Pictures represent mature flowers under control conditions (**a**) and high temperature (**b**–**c**). Under high temperature, anthers showed deformation, dark coloration of the anther tip and elongated pistils. Those flowers had a low percentage of pollen viability (<10%).

(i) The alteration of membrane structures under high temperature is a common response to heat stress. High temperatures alter membrane permeability by increasing membrane fluidity. This leads to an increase of electrolyte loss which is an indication of decreased cell membrane stability [3].

(ii) Photosynthesis is highly sensitive to high temperature [6]. The alteration of the thylakoid membrane under heat stress is directly linked to a decrease of photosystem II activity which affects the rate of photosynthesis [7]. In addition, the sensitivity of the enzyme Rubisco to high temperature can lead to a decrease in carboxylase activity which will also inhibit photosynthesis [8] and likewise CO_2_ fixation. Consequently, the ability to assimilate CO_2_ under heat stress conditions is associated with thermo-tolerance.

(iii) Reproduction has been demonstrated as the most heat sensitive process in plants as reviewed in two recent papers [9,10] (Figure 2). Many studies have shown that pollen quality is the most important determinant of fruit production under heat stress [11,12]. Therefore, analysis of pollen viability and fruit set under high temperature is a direct trait for the study of thermo-tolerance in plants.

Heat stress induces a reorganisation of the transcriptome [13], proteome [14] and metabolome [15] which can either lead to failures in plant development, or instead to acclimation to high temperature [9]. On the one hand, metabolic alterations caused by heat stress can lead to severe damage in sensitive plants compared to tolerant plants. For example, high temperature can lead to a decrease of antioxidant enzyme activity [16]. This will decrease the plant’s ability to protect itself against reactive oxygen species and leads to lipid peroxidation of cellular membranes. In addition, the alteration of photosynthesis activity under high temperature can lead to a decrease of sugar abundance [17]. Sugars are primary metabolites essential as precursors for different metabolic pathways and plant nutrition. On the other hand, plants have the ability to respond to heat stress by inducing or activating protective mechanisms. For example, most of the transcripts induced in response to high temperature encode heat shock proteins (HSPs) [18]. HSPs are proteins which are rapidly produced under heat stress and behave as protein chaperones [19]. HSPs are involved in protein homeostasis in order to avoid protein misfolding, protein aggregation or protein degradation. They are closely linked with thermo-tolerance and play a crucial role in stress signal transduction [20]. In addition to HSPs, plants can produce different osmolytes and antioxidants to protect themselves from various abiotic stresses, including heat stress [21]; Proline, glycine betaine and aminobutyric acid are key compounds in the osmolyte response under high temperature [22,23,24]. Carotenoids, glutathione and ascorbate can act as reactive oxygen species scavengers in order to prevent oxidative stress [25,26,27]. So, the readjustment of cellular homeostasis under heat stress is an essential mechanism which can provide resistance to high temperature and the ability to produce fruit under suboptimal conditions. 

In this review, we will focus on the impact of heat stress on the metabolome of pollen (one of the most sensitive organs in plants). We will first review the metabolic changes occurring during pollen development. Secondly, we will describe the impact of heat stress on pollen quality and the metabolic changes associated with thermo-tolerance. Finally, we will discuss various breeding strategies for pollen thermo-tolerance based on the use of metabolic markers.

## 2. Metabolite Profiles during Pollen Development

**Figure 3:**
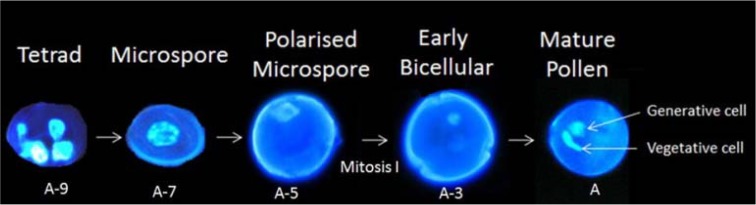
Pollen development from tetrad stage to mature pollen stage. Days before anthesis related to developmental stages are based on *Lycopersicon esculentum* Mill. “Trust” from [32] and [33]. **A**, anthesis, **A-3**, 3 days before anthesis, **A-5**, 5 days before anthesis, **A-7**, 7 days before anthesis, **A-9**, 9 days before anthesis. Nuclei are stained with DAPI.

Pollen is the male gametophyte and its role is to deliver the genetic material to the embryo sac through the double fertilisation as described by Twell, 2002 and Honys *et al.*, 2006 [28,29]. The development of the pollen takes place inside the anthers (Figure 3). In early stages, the anthers have several sporogenous layers. These primary layers lead to the development of meiocytes which occur inside the loculus within the anthers. The loculus is surrounded by the tapetum which provides nutrition, metabolites and enzymes required for the development and the protection of the pollen before it degenerates during mitosis. Meiocytes undergo meiotic division to produce haploid tetrads. The four microspores of the tetrad are released by the action of a glucanase which is produced by the tapetum. At the microspore stage, the pollen coat is synthesised. The pollen wall consists of two layers; an outer layer named the exine, which is composed of sporopollenin and shows apertures where the pollen can germinate; the internal layer is the intine, composed of pectin and cellulose. The pollen coat plays a protective role during pollen dispersion and can also play a role in the attachment to a pollinator. Microspores undergo mitotic divisions to produce the mature pollen, which are composed of a vegetative nucleus and two sperm cells. Between the microspore stage and the mature stage, a phase of vacuolisation occurs which leads to an increase of the pollen size and a polarisation of the nucleus [30]. At mature stage, the pollen dehydrates which provides a level of tolerance to environmental stresses [31]. Rehydration of the pollen happens on a compatible pistil and leads to germination and growth of the pollen tube inside the pistil to deliver the male gamete for the double fertilisation. 

### 2.1. Carbohydrates and Acid Invertase

Carbohydrates are important substrates for plant growth which can be stored as energy reserves and also serve as signalling molecules [34]. Male sterile lines of Indian mustard showed an altered sugar metabolism in reproductive tissue, suggesting a causal relationship between sugar metabolism and fertility [35]. Pacini, 1996 [36] reviewed carbohydrate reserves in pollen of different species. The main soluble sugars in mature pollen are fructose, glucose, sucrose and starch. Sucrose is produced in photosynthetic tissue such as leaves which is considered as source tissue from which sucrose is transported via the phloem to sink tissue [37]. Pollen is considered a sink tissue which needs sugars for its own development, growth and protection against environmental stresses. 

The conversion of sucrose into hexoses, which can be used by the pollen, requires its hydrolysis by acid invertase and/or sucrose synthase [38]. Koch, 2004 reviewed the role of acid invertase in plants; Three different acid invertases can be identified according to their location: vacuolar invertase (VIN), cytoplasmic invertase (CIN) and cell wall invertase (CWIN). The function of acid invertases in reproductive tissues has been intensely studied in different species such as lily [39,40], tobacco [41,42], tomato [33] and chilli pepper [43].

The physiology of carbohydrate accumulation during anther and pollen development has been studied in lily (*Lilium auratum*) [44,45]; Clément and Audran, 1995 [44], showed that, in lily, the different cells which constitute anthers are connected via plasmodesmata involved in symplasmic transport of carbohydrates from the phloem to the internal anther layers. However, the transport of assimilates from internal anther layers to the pollen follows an apoplastic pathway. The main carbohydrates found in lily anthers were sucrose, glucose, fructose and starch [45]. During the anther growth, starch was used and this decrease correlated with a strong accumulation of soluble sugars. This was also found by Pressman* et al.*, 2002 in tomato [33]. In addition, Castro and Clément, 2007 [40] studied the content of carbohydrates in different fractions of lily anthers, such as anther wall, locular fluid and pollen. The accumulation of soluble sugars in the locular fluid suggested that this tissue may play a role in sugar storage during pollen development, which is especially useful once the tapetum is degraded. In lily, soluble sugars also accumulated in mature pollen, mainly in the form of hexoses, while in tomato sucrose represented 80% of the carbohydrate fraction in mature pollen [46]. Although we cannot exclude that the differential accumulation of hexoses *vs.* sucrose may be species specific, Castro and Clément, 2007 [40], notified that the lily pollen used for sugar analyses were hydrated. Using tomato pollen, we observed that hydrated pollen grains have a higher abundance of hexoses than dried pollen grains, due to the action of acid invertase upon (re-)hydration. Therefore, if the pollen isolation protocol requires immersion into a germination solution, we strongly advise to freeze-dry the pollen extract as soon as the harvest is done to prevent the action of acid invertase which may convert sucrose into hexoses, or to incubate the extract in 80% ethanol at 75 °C as it was previously described by Pressman* et al.*, 2012 [46] and Firon* et al.*, 2006 [12]. Nevertheless, Castro and Clément, 2007 found a gradient of soluble sugars from the anther to the pollen underlying the strong sink of the pollen [40]. During pollen maturation in tomato, sucrose was the most abundant sugar in the filament and in the pollen grain, whereas hexoses were the most abundant sugars in the anther wall and in the locular fluid [46]. The higher abundance of hexoses in the anther wall was supported with a high activity of acid invertase and a low activity of sucrose phosphate synthase. The accumulated hexoses in anther wall could be used for general metabolism or could move into the locular fluid. The distribution of hexoses among the different stamen parts were mainly controlled by cell wall acid invertase. The high accumulation of sucrose in mature pollen could serve as energy source for pollen germination, but could also act as osmolyte to maintain membrane integrity during pollen dehydration [46]. To summarize, soluble sugars can be delivered to the pollen by (i) hydrolysis of starch (ii) transport of sucrose from photosynthetic tissues. The sucrose needs to cross several compartments before it reaches the pollen in order to provide nutrition and protection: sucrose has to be transported from the leaves via the phloem to reach the anther wall or directly coming from the anther itself [47]. Subsequently, it has to cross the locular fluid to reach the male gametophyte. Acid invertases play a major role in supplying hexoses, derived from sucrose, to the pollen grain to support nutrition, growth and protection against environmental stresses. 

### 2.2. Proline

Amino acids play an essential role during plant development: they are the building blocks for protein synthesis. Proline is a free amino acid synthetized from glutamate, which can act as a compatible solute to achieve osmotic adjustment [48,49]. Proline is one of the most abundant amino acids in the male reproductive part [50]. The changes in amino acid content during pollen development in anthers has been studied in devil’s trumpet (*Datura metel)*, a *Solanaceous* species [51]. The content of free amino acids in pollen was higher at mature stage compared to earlier developmental stages. It was suggested that the lower level of amino acids during early developmental stages was due to active protein synthesis at these stages. The accumulation of glutamic acid decreased during pollen development, probably due to conversion into proline. Proline was one of the most abundant amino acids at mature stage and represented 60% of the free amino-acids. In arabidopsis (*Arabidopsis thaliana*), a proline deficient mutant showed a decreased pollen viability which indicates a role for proline in pollen development. The proline mutant could be partially complemented by spraying proline on the mutant inflorescences. This led to an improvement of pollen germination [52].

### 2.3. Lipids

In contrast to carbohydrates, the role of lipids during pollen development has not been studied extensively. Lipids are important compounds for the stabilization of membranes and for the hydration of pollen upon germination [53]. Rodriguez-Garcia* et al.*, 2003 [54], showed that lipids accumulated during pollen development in olive (*Olea europaea*). The accumulation of lipids occurred after the vacuolisation of the microspore. During pollen germination, lipids are located close to pollen apertures. This suggests that lipids may act as energy reserve for pollen germination. The lipid profile of pollen at different developmental stages has been studied in trumpet vine (*Campsis radicans*) (Bignoniaceae) [55]. Until the microspore mother cell stage, anthers contained low levels of lipids and a high abundance of insoluble polysaccharides. At meiosis, insoluble polysaccharides decreased whereas lipids started to accumulate. The authors suggested that anthers switched from the use of polysaccharides to lipids as storage products. Additionally, lipids have been showed to play a major role in directing the pollen tube to the stigma [56]. 

### 2.4. Gluthatione

Glutathione is a thiol which participates in redox regulation and plays a role in storage, transport, and regulation of metabolites. Furthermore, it is involved in the detoxification of reactive oxygen species [57]. The involvement of glutathione in redox regulation plays a crucial role in withstanding environmental stresses [58]. The application of buthionine sulfoximine, an enzyme inhibitor in the glutathione pathway, led to a 70% decrease of* in vitro* pollen germination in arabidopsis [59] demonstrating the crucial role of glutathione in pollen germination.

### 2.5. Flavonoids

Secondary metabolites produced in the tapetum, such as phenolic compounds, can diffuse to the pollen and play a role in pollen colour, in the attraction of pollinators, in pollen tube germination and in protection against abiotic stress of pollen. Flavonoids can be grouped into different classes, such as flavonols, flavones, isoflavones and anthocyanins. Antioxidant flavonoids play a role in plant development as ROS scavengers and may act as regulators under environmental stress [60,61]. Addition of flavonols to germination medium resulted in higher levels of* in vitro* germination of tobacco (*Nicotiana tabacum*) pollen [62]. Down-regulation of chalcone synthase (*CHS*), the first step in the flavonoid pathway, led to a decrease of flavonoids and subsequently to a reduction of pollen germination in petunia (*Petunia hybrida*) and maize (*Zea mays*). The germination could be restored by adding kaempferol to the germination medium [63]. A confirmation of the importance of flavonoids in pollen fertility has been given by Napoli* et al.*, 1991 [64] who complemented a petunia *chs* mutant with a functional *CHS* transgene, thereby restoring the fertility of petunia pollen. In addition, RNAi-mediated silencing of *CHS* in tomato resulted in male sterility, reduced fruit set and seed-less fruit development [65]. In arabidopsis, however, a mutation of CHS (tt4) did not affect pollen germination [66]. The role of flavonoids in pollen fertility is therefore species specific. 

### 2.6. Polyamines

Polyamines, such as putrescine, spermidine and spermine are synthetized from ornithine, arginine or methionine. They play a role in different aspects of plant development such as cell division, embryogenesis, root development, floral initiation, floral and fruit development and also pollen formation [67]. In tobacco pollen, the polyamine content increased during development from microspore to mature stage, but decreased in germinated pollen, probably due to their consumption during the germination process [68]. *Song et al.*, 2001 [69] showed that, despite the decrease of polyamines in germinated pollen, a transient increase of spermidine and spermine at the beginning of pollen tube growth was needed for normal pollen germination in tomato. In the pollen of kiwi (*Actinidia deliciosa*), polyamines were present from early stage (microspore) to mature pollen with spermidine as the most abundant polyamine. Adding polyamine inhibitors led to abnormal pollen development and a reduction in pollen viability, pollen germination and pollen tube growth [70]. 

### 2.7. Hormones

Hormones are essential in plant development; they play a role in the regulation of flowering time, leaf senescence, fruit ripening and also pollen development. *Parish et al.*, 2013 [71], underlined the role of auxin, gibberelins and abscisic acid in the development of the tapetum which is essential for the distribution of metabolites to the pollen. In addition, there is accumulating evidence for a role of other hormones, such as ethylene, jasmonic acid and brassinosteroids in pollen development. The current state of the art will be outlined below.

Auxin is involved in many aspects of plant development, such as plant growth, senescence, fruit formation, leaf abscission and apical dominance [72,73,74,75]. At the genetic level, the biosynthetic pathway to auxin remains unclear and up to five possible pathways have been postulated [76]. One of the evolutionary most conserved auxin pathways, the indole-3-pyruvic acid (IPA) pathway, is controlled by members of the *YUCCA* flavin monooxygenase gene family. Blocking the auxin biosynthesis pathway in arabidopsis by various combinations of *yuc* loss-of-function mutants led to severe alterations in floral organ development and a lack of pollen production [77]. In addition, mutations in auxin receptor encoding genes, such as *transport inhibitor response 1* (*tir1)* and *auxin signalling F box (afb)* genes, resulted in early maturation of pollen due to pollen release before the filament was completely elongated [78,79]. Cecchetti* et al.*, 2013 also proposed that auxin is involved in the coordination of pollen maturation and anther dehiscence. This is supported by the fact that expression of the indole acetic acid lysine synthetase (*iaal*) gene in anther tapetum of transgenic tobacco plants led to a decrease of auxin levels and a concomitant decrease in *in vitro* pollen embryogenesis [80]. Culture medium supplemented with auxin restored pollen embryogenesis in transgenic *iaal* plants. 

Gibberellins (GA) are known to act in hypocotyl elongation, floral transition, fruit patterning and plant defence [81]. *ga1-1*, an arabidopsis mutant deficient in gibberellin production resulted in an inhibition of stamen elongation and lack of mature pollen. This phenotype could be restored by adding exogenous gibberellins [82]. The mutants GA-deficient *rpe1* and GA-insensitive *slr1-3* in rice are male sterile. *rpe1* was defective in pollen germination and elongation whereas *slr1-3* affected pollen development [83]. 

Jasmonic acid plays a role in fruit ripening, seed germination, root growth, resistance to biotic stresses and protein storage [84]. The role of jasmonic acid in pollen fertility has been studied by McConn and Browse, 1996 [85]. A mutation in the biosynthesis of jasmonic acid, *defective in anther dehiscence1* (*dad*1), led to an inhibition of pollen release. The *dad*1 mutation also led to a 68% decrease of pollen germination after manual pollen release. Pollen germination could be recovered by addition of jasmonic acid [86]. 

Ethylene is known to be involved in plant development, senescence, fruit maturation and pollen germination [87]. An inhibitor of ethylene action (NBD) demonstrated the role of ethylene in pollen development in petunia [88]. Two ethylene peaks were observed during pollen development, at microspore development stage and at maturation. Exogenous NBD completely inhibited anther development at early developmental stages and delayed anther dehiscence. In tobacco, moreover, an ethylene-receptor mutant showed a delay in anther dehiscence compared to the wild-type plants [89]. Ethylene, therefore, plays a role in locule opening. 

Abscisic acid is important in seed development, plant growth and in withstanding environmental stresses [90]. Frascaroli* et al.*, 1993 [91] showed that a low level of abscisic acid (ABA) in the germination medium increased the germination of maize pollen whereas a higher level decreased germination. In pomegranate (*Punica granatum*), Yang* et al.*, 2003 [92] also noticed that a high concentration of ABA in the germination medium decreased pollen germination. However, a low exogenous ABA concentration in the germination medium did not improve pollen germination. Nevertheless, inhibition of endogenous ABA biosynthesis led to a decrease of pollen germination and it was concluded that a minimal ABA concentration was needed for pollen germination.

Brassinosteroids are steroid hormones which play a role in plant growth and several mutants of the brassinosteroid pathway show abnormal growth characteristics [93]. An arabidopsis mutant, *transient defective exine 1* (*tde1*) is deficient in the formation of the pollen exine, which is essential for protection and propagation of pollen. The mutated gene has a high similarity to a gene involved in the biosynthesis of brassinosteroids and supplementation with brassinosteroids led to restoration of exine layer formation [94]. 

**Figure 4:**
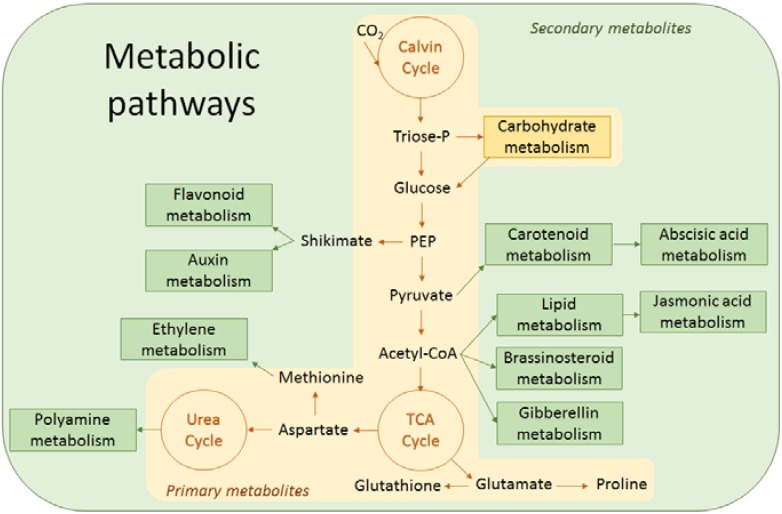
Simplified metabolic pathways underlying the relationship between metabolites reported in this review such as carbohydrates, proline, lipids, glutathione, polyamines, flavonoids and hormones. PEP, phosphoenolpyruvate; TCA, tricarboxylic acid. Triose-P, triose-phosphate.

During pollen development, several metabolic changes occur leading to the accumulation of reserves necessary for nutrition and protection of the pollen (Table 1). The pathways leading to the production of carbohydrates, amino acids, phenolic compounds, polyamines, hormones and lipids are interconnected and contribute to metabolic homeostasis required for growth and viability of the pollen (Figure 4). This metabolic equilibrium is sensitive to environmental stresses. The effect of heat stress on pollen development and fertility is likely resulting from an altered metabolic homeostasis, caused by alterations in the levels and composition of the above mentioned metabolites. This will be discussed in detail in the next sections. 

**Table 1:** Overview of the role of different metabolites in pollen development and fertility among different species.

Commun name	Latin name	Metabolites	Implication	References
arabidopsis	*Arabidopsis thaliana*	Proline	Required for pollen germination	[52]
		Glutathione	Required for pollen germination	[59]
		Auxin	Required for floral organ development and pollen production	[77]
			Required for pollen maturation and anther dehiscence	[79]
		Gibberellin	Required for stamen elongation and pollen maturation	[82]
		Jasmonic acid	Required for pollen germination	[85,86]
		Brassinosteroid	Formation of pollen exine	[94]
		Flavonoids	Not required for pollen germination	[66]
Devil’s trumpet	*Datura metel*	Amino acids	Accumulation during pollen development	[51]
			Proline represents 60% of the free amino-acids	
kiwi	*Actinidia deliciosa*	Polyamines	Required for pollen viability and pollen germination	[70]
lily	*Lilium auratum*	Carbohydrates	Accumulation of soluble sugars during anther development	[45]
			Locular fluid is a sugar storage compartment	[40]
			Gradient of sugars from anther wall to pollen grain	
maize	*Zea mays*	Flavonoids	Required for pollen germination	[63]
		Abscisic acid	High ABA concentration inhibited *in-vitro* pollen germination	[91]
olive	*Olea europaea*	Lipids	Accumulation of lipids during pollen development	[54]
			Energy reserve for pollen germination	
petunia	*Petunia hybrida*	Flavonoids	Required for pollen germination	[63,64]
		Ethylene	Required in anther development and anther dehiscence	[88]
pomegrenate	*Punica granatum*	Abscisic acid	High ABA concentration inhibited *in-vitro* pollen germination	[92]
rice	*Oryza sativa*	Gibberellin	Required for pollen germination, stamen elongation and pollen development	[83]
tobacco	*Nicotiana tabacum*	Flavonoids	Improve *in-vitro* pollen germination	[62]
		Polyamines	Accumulation of polyamines during pollen development	[68]
			Reduction of polyamines during pollen germination	
		Auxin	Required for pollen embryogenesis	[80]
		Ethylene	Control anther dehiscence	[89]
tomato	*Solanum lycopersicum*	Carbohydrates	Increase of soluble sugars during pollen and anther development	[33]
			Sucrose represents 80% of total carbohydrates in pollen mature	[46]
		Flavonoids	Required for pollen fertility and fruit set	[65]
		Polyamines	Accumulation of spermidine and spermine is required for pollen germination	[69]
trumpet vine	*Campsis radicans*	Lipids	Accumulation of lipids during anther development	[55]

## 3. The Impact of Heat Stress on Pollen Quality

### 3.1. Pollen Viability

Pollen is one of the most sensitive organs to heat stress. This can lead to sterile pollen [95] and subsequently a decrease of fruit set [96]. As a consequence, crop yield is dependent on the temperature during pollen development [97]. In tomato, the optimum temperature is between 18 °C and 25 °C [98]. A few degrees higher than 25 °C already leads to a reduction of yield [99]. The impact of heat stress on pollen viability has already been demonstrated in several species: tomato [12,33,96,99,100]; barley (*Hordeum vulgare*) [101], rice [102,103], chickpea (*Cicer arietinum*) [104], maize [105], arabidopsis [106], rapeseed (*Brassica napus*) [107], purple false brome (*Brachypodium distachyon*) [108], common bean (*Phaseolus vulgaris*) [109], groundnut (*Arachis hypogaea*) [110], bell pepper (Capsicum annuum) [111], soybean (*Glycine max*) [112], chickpea (*Cicer arietinum*) [104,113] and strawberry (*Fragaria x ananassa*) [114].

### 3.2. Pollen Development

At early stages of pollen development, high temperatures lead to an arrest in pollen development [10]. In tomato, the most sensitive stage is between 10 and 7 days before anthesis [115]. This was confirmed by Sato* et al.*, 2002 [116], who showed that a temperature regime of 32 °C/26 °C, 15–7 days before anthesis, corresponding to the stage in which meiosis takes place, had a profound effect on pollen development. Heat stress during meiosis also has a large effect in barley [101]. In cowpea (*Vigna unguiculata*), however, heat stress during meiosis did not affect pollen quality, but when it was too hot after the release of tetrads (between 9 and 7 days before anthesis) an effect on pollen quality was observed [117]. In peanuts, the most sensitive stages were during the development of microspores (four days before anthesis) and at anthesis [118]. In bell pepper, heat stress at microspore mother cell meiosis led to reduction of pollen viability, fruit set and seed number, whereas heat stress at later developmental stages did not affect the pollen viability [119]. The developmental sensitivity to heat stress in pollen seems to be species-specific, with meiosis a common sensitive stage, for most crops. 

### 3.3. Tapetum

The tapetum is the key organ that provides metabolites to the pollen; its development is very sensitive to heat stress. In barley, for example, an elevated temperature of 30 °C led to an early meiotic prophase I and a premature degradation of the tapetum [120]. A degeneration of the tapetum under heat stress has also been reported in wheat [95], cowpea [117], purple false brome [108] and common bean [121]. Suzuki* et al.*, 2001 [121] showed that heat stress 10 days before anthesis affected the endoplasmic reticulum pattern of the tapetum showing structural abnormalities which subsequently led to a premature degeneration of tapetum. In summary, it is clear that the development of the tapetum is critically sensitive to heat stress and can lead to abnormal anther development. In addition, heat-stress induced abnormalities in tapetum development will also affect the provision of metabolites to the pollen, which can lead to severe effects on pollen nutrition. 

### 3.4. Opening of Loculi

**Figure 5:**
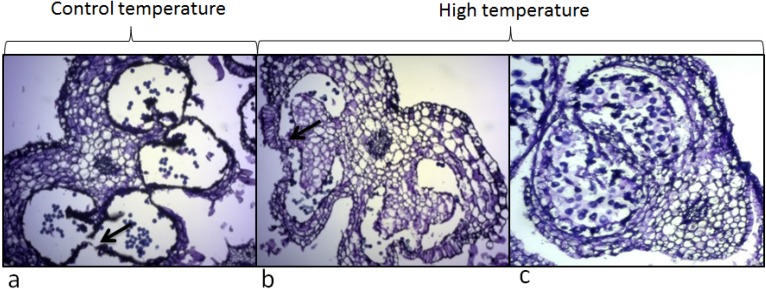
Effect of high temperature (32 °C/26 °C) on anthers of *Solanum lycopersicum* cv. microTom. Picture (**a**) shows an anther at mature stage of pollen development under control conditions. The opening of the locule is indicated with an arrow. Picture (**b**) shows anther at mature stage of pollen development under high temperature. The locule was not opened due to anther deformation and the presence of an extra layer of cells. Picture (**c**) shows a severe anther deformation under high temperature. The four distinct locules were no longer visible.

In rice, heat stress affected the opening of the anther loculi which led to a decrease of pollen fertility [103]. The same result was also observed in tomato (Figure 5). An inhibition of anther loculi opening may therefore block the release of pollen grain. 

### 3.5. Pollen Germination

In addition to pollen development, pollen germination is sensitive to heat stress, which can prevent pollen tube growth. Hazra* et al.*, 2008 [122] established that the decrease of fruit set of tomato under elevated temperature was mostly due to a reduction of pollen germination. Reduction of pollen germination under heat stress can be due to earlier pollen development failure. High temperatures also led to a decrease of* in vitro* pollen germination in cotton (*Gossypium hirsutum*) [123]. The pollen germination starts to decrease at 30 °C (tomato: [124] cucurbita (*Cucurbita pepo*): [125], cotton: [123]). Pollen germination was altered in tomato under heat stress [126] and led to a delay in fruit development and parthenocarpy [127,128]. Plants that can be potentially parthenocarpic become visible when no viable pollen are present; this has been observed in tomato, bell pepper [111,119] and in rapeseed [107]. 

## 4. Impact of Heat Stress on Pollen Metabolites

### 4.1. Carbohydrates

The importance of carbohydrates for plant homeostasis is reflected on the one hand by their function as energy source required for pollen development and germination and, on the other hand, by their role in maintaining osmotic balance, in stress signalling and in protecting membranes. As a consequence, a disruption in carbohydrate metabolism may affect plant nutrition as well as protection against stresses, in both cases leading to a decrease in plant performance and health. The influence of abiotic stresses such as cold, drought and heat on sugar signaling has been recently reviewed [10]. The impact of heat stress on pollen carbohydrates has been studied in tomato, chickpea, pepper and sorghum (*Sorghum bicolor*) over the last years. In tomato, flowers developed under an elevated temperature of 32 °C showed a decrease of pollen viability. These flowers showed a decrease of soluble sugars in pollen and anther wall, but an increase of soluble sugars in the locular fluid [33]. Pressman* et al.*, 2002 suggested that since under high temperature pollen was altered, the pollen’s need for soluble sugars was decreased, and therefore soluble sugars were accumulated in the locular fluid. However, we can also speculate that under high temperature soluble sugars are blocked into the locular fluid and cannot reach the pollen. The authors also found that during normal pollen development, starch accumulated in pollen reaching a maximum content three days before anthesis and from this moment onwards starch was converted into soluble sugars. However, under hot conditions, starch concentration did not increase and led to a lack of sugar conversion, explaining the decrease of soluble sugars in mature pollen grains. This indicates that mild chronic heat stress leads to alterations in sugar transport and/or metabolism and an altered distribution of soluble sugars over the different anther tissues. In another study, it was shown that the activity of acid invertase in tomato flowers that were, at 4 days after anthesis, exposed to high temperature (36 °C/28 °C) for 24 h was lower in a sensitive genotype than in a thermo-tolerant genotype. High acid invertase activities may, therefore, play a role in producing hexoses under stress conditions [129]. The effect of heat stress on soluble sugar abundance may be an important factor contributing to the decrease of pollen viability, since thermo-tolerant tomato genotypes, showing high pollen viability under long heat stress exposure (32 °C/26 °C), did not show heat stress-induced sugar alterations compared to thermo-sensitive genotypes [12]. Alteration of carbohydrate accumulation under high temperature would probably lead to a decreased availability of energy resources and a decrease in the osmotic power of carbohydrates, leading to a failure in pollen development. These results are supported by studies in sorghum in which the decrease of pollen viability under long heat stress (36 °C/26 °C) was mainly correlated with a decrease of starch and sucrose in late stages of pollen development, due to decreased expression of several sugar metabolism genes [130]. In chickpea, sensitive genotypes had also a lower abundance of sugars in anthers and pollen than tolerant genotypes in a warm season (above 32 °C/20 °C) [113]. The low abundance of sugars was due on one hand to a decrease of sucrose metabolism in leaves and on the other hand to a decrease of sucrose synthase and acid invertase enzymes in anthers. Under high temperature, hexose abundance increased in tolerant genotypes whereas it decreased in sensitive genotypes. The increase of hexoses may, therefore, have a protective role under high temperatures. A contrasting effect was seen, in bell pepper pollen, where sucrose levels increased rather than decreased when plants were, at eight days before anthesis, exposed to high temperature (32 °C/26 °C) until they reached anthesis. This was mainly due to a decrease of acid invertase and hexokinase concentrations [111,131]. Despite the high levels of sucrose, pollen viability decreased under high temperatures. This suggests that, at least in this bell pepper variety, high levels of sucrose are not sufficient to provide a normal pollen development under high temperature. The contrasting results found in bell pepper compared to the other examples described above could be attributed to the different developmental stages at which the heat stress was applied. In conclusion, activities of acid invertases and the abundance of soluble sugars and starch play an important role in maintaining pollen quality under high temperature. 

### 4.2. Proline

Proline is a common amino acid which can accumulate in response to various environmental stresses. Proline has been shown to play a role in the protection of membrane integrity, in ROS scavenging and in maintaining cellular homeostasis [22,49]. Incubation of pollen extract in germination medium at 40 °C during 10 min decreased* in vitro* germination of lily pollen and this could be restored by adding proline to the medium [132]. The hypothesis is that proline can stabilise proteins by maintaining the hydration shells around molecules. In cowpea, the content of proline was analysed in anthers of heat sensitive and heat tolerant cultivars developed under high temperature (the maximum temperature per day was 45 °C). The mature pollen of the tolerant cultivars had a higher abundance of proline compared to sensitive pollen, while heat sensitive cultivars accumulated the highest proline levels in anthers [50]. The authors concluded that in heat sensitive cultivars the transfer of proline from anthers to pollen was inhibited. The impact of heat stress on proline transport was confirmed by Sato* et al.*, 2006 [100] who showed that the expression of the *proline transporter 1* gene decreased in anthers of tomato under long heat stress (32 °C/26 °C). In contrast, in rice the proline content decreased in anthers of plants exposed to 39 °C for 4 h per day during 5 days. This might be caused by another mechanism involving the transport of proline from the vegetative part to the reproductive part of the plant [133].

### 4.3. Lipids

Lipids play an important role in membrane fluidity especially under stress conditions. In sorghum, a high temperature of 32 °C/28 °C for 10 days led to an increase of ROS content and membrane alterations in pollen [134]. This increase was correlated with a decrease of pollen viability and an alteration in pollen phospholipid content. Under high temperature the abundance of bound unsaturated fatty acids increased whereas the abundance of saturated fatty acids decreased. The authors suggested that membrane damage caused by high temperature could be resulting from the increase of unsaturated fatty acids which will lead to an increase of membrane fluidity due to the presence of double bonds. The unsaturated fatty acid moieties make the membrane more vulnerable to ROS attacks. Similar results were found in soybean from which the decrease of pollen viability was also correlated to an alteration of in phospholipid saturation [112].

### 4.4. Polyamines

Polyamines have been reported to act in tolerance to many stresses such as cold, heat, salt, drought and high metal concentrations [135,136]. They can act as ROS scavengers and maintain membrane integrity [137]. In tomato, incubation of pollen extract in germination medium for 20 h at 33 °C decreased the* in vitro* pollen germination and this could be reversed by adding spermidine or spermine to the medium [138]. This observation suggested that heat stress decreased the level of polyamines. Indeed, an incubation of pollen at 38 °C during 4 h lowered the content of spermidine and spermine whereas the content of putrescine increased and these changes correlated with a decrease of pollen germination [139]. Adding spermidine or spermine to the medium restored the level of pollen germination. The change in polyamine content and the decrease of pollen germination was mostly due to a decrease of SAMDC (S-adenosylmethionine decarboxylase) under heat stress. Blocking SAMDC translation with cycloheximide could phenocopy the effect of heat stress, leading to decreased pollen germination under control conditions. However, we cannot exclude that other proteins affecting pollen germination may have been inhibited by cycloheximide as well. Contrasting results were found in Japanese apricot (*Prunus mume*), in which a heat stress of 24 h at 35 °C decreased pollen germination, but this could not be restored by adding polyamines to the medium. It was hypothesised that this may have been due to a toxic level of exogenous polyamines [140]. Nevertheless, results in many crops suggest that polyamines have an important role in pollen germination under heat stress.

### 4.5. Hormones

Brassinosteroids are steroidal hormones which play a role in responses to various abiotic stresses, such as heat, salt and drought stress. They interact with heat shock proteins and also play a role in ROS scavenging [141]. Under heat stress (35 °C for 4 h), the* in vitro* pollen germination of tomato was increased in the presence of 24-epibrassinolide, a brassinosteroid [142], demonstrating a role for brassinosteroids in pollen germination under high temperatures. 

Ethylene is also involved in the response to heat stress [143]. A mutation of the ethylene receptor (*nr*) increased the number of non-viable pollen under long heat stress (32 °C/26 °C) and treatment of the plants with ethylene increased pollen viability under long heat stress [144].

A role for auxin under environmental stresses has been demonstrated in rice under drought conditions [145] and several genes involved in the auxin pathway are known to have a role in withstanding abiotic stress [146]. Under heat stress (30 °C/25 °C for 5 days), the level of auxin decreased in anthers of barley and arabidopsis, which correlated to male sterility [147]. The application of exogenous auxin reversed male sterility. Auxin content decreased in anthers of rice plants exposed to 39 °C for 4 h per day during 5 days and was correlated with a decrease of pollen viability and pollen germination [133]. In this same study, the content in abscisic acid (ABA) and gibberelins (GA) was also analysed. GA concentrations decreased under high temperatures and ABA concentrations increased. High levels of ABA were already known to be associated with a decrease of pollen germination [91].

**Figure 6:**
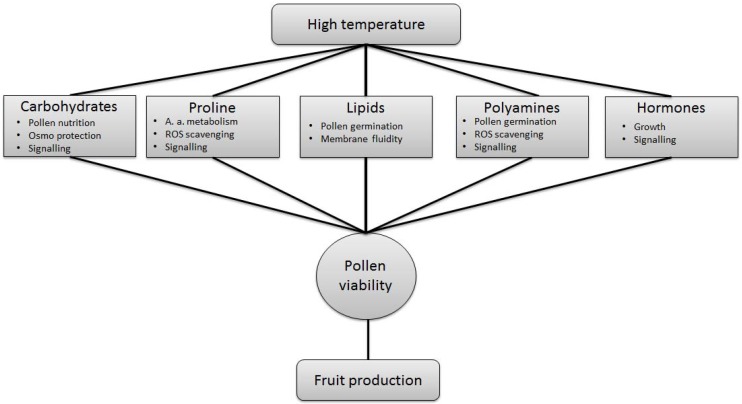
Metabolites affected by heat stress and their role in providing viable pollen.

To tolerate heat stress, plants have to maintain metabolic homeostasis in order to avoid disruption of pathways which could lead to abnormal accumulation or reduction of compounds essential for pollen development (Figure 6). A high temperature can affect several compounds at the same time; however, most of the studies are based on the analysis of one group of compounds only (Table 2). Analysis of metabolites in pollen with untargeted approaches could provide a global view on the metabolic responses to heat stress and may lead to the identification of additional, novel compounds involved in the response to heat stress and thermo-tolerance. 

**Table 2:** Summary table of the impact of high temperature on the pollen metabolome in different species. HS, heat stress.

Common name	Latin name	Metabolites	Temperatures	Heat-stress length	Plant material used	Impacts	References
tomato	*Solanum lycopersicum*	Carbohydrates	32 °C/26 °C	Four months	Flowers totally developed under HS	Decrease in pollen mature	[33]
			32 °C/26 °C	Three months	Flowers totally developed under HS	Sugar abundance decreased in thermo-sensitive genotype compare to thermo-tolerant ones	[12]
			36 °C/28 °C	24 h	Plants at four days after anthesis	Decrease of acid invertase activity in flowers of sensitive genotype compare to tolerant genotype	[14]
		Proline	32 °C/26 °C	Long	Flowers totally developed under HS	Proline transporter expression decreased in anthers	[100]
		Polyamines	33 °C, 35 °C, 38 °C	20 h	Pollen germination medium	Adding polyamines improved* in-vitro* pollen germination	[139]
			38 °C	4 h	Pollen germination medium	Decrease in pollen mature	[138]
		Brassinosteroids	35 °C	4 h	Pollen germination medium	Adding brassinosteroids improved* in-vitro* pollen germination	[142]
		Ethylene	32 °C/26 °C	Three months	Flowers totally developed under HS	Ethylene transporter mutation decreased pollen viability	[46]
barley	*Hordeum vulgare*	Auxin	30 °C/25 °C	Five days	Plants at five leaf stage	Decrease in anther and exogenous auxin restored male sterility	[147]
rice	*Oryza sativa*	Proline	39 °C	4 h per day during five days	Plants at flowering stage	Decrease in anther	[133]
		Auxin	39 °C	5 h per day during five days	Plants at flowering stage	Decrease in anther	[133]
		Gibberelin	39 °C	4 h per day during five days	Plants at flowering stage	Decrease in anther	[133]
		Abscisic acid	39 °C	4 h per day during five days	Plants at flowering stage	Increase in anther	[133]
arabidopsis	*Arabidopsis thaliana*	Auxin	30 °C/25 °C	Five days	Plants at five leaf stage	Decrease in anther and exogenous auxin restored male sterility	[147]
bell pepper	*Capsicum annuum*	Carbohydrates	32 °C/26 °C	For eight days before anthesis	Plants at flowering stage	Increase of sucrose due to a decrease of acid invertase activity	[111]
sorghum	*Sorghum bicolor*	Carbohydrates	36 °C/26 °C	Long-season	Flowers totally developed under HS	Decrease of sucrose and starch in pollen mature	[130]
		Lipids	32 °C/28 °C	Ten days	Plants at 40 days after sowing	Decrease of saturated fatty acids which weaken the membrane to ROS attack	[134]
chickpea	*Cicer arietinum*	Carbohydrates	Above 32 °C/20 °C	Long	Flowers totally developed under HS	Decrease of sugars in sensitive genotypes compare to tolerant genotype in anthers. Tolerant genotype increased hexose abundance in anthers compare to sensitive genotype.	[113]
lily	*Lilium auratum*	Proline	40 °C	10 min	Pollen germination medium	Adding proline improved *in-vitro* pollen germination	[132]
cowpea	*Vigna unguiculata*	Proline	45 °C/25 °C	Long	Flowers totally developed under HS	Proline accumulated in anther wheareas in pollen it decreased. Tolerant genotype had a higher abundance of proline in pollen	[50]
soybean	*Glycine max*	Lipids	From 30 °C/23 °C to 39 °C/20 °C	Ten days	Flowering stage	Decrease of saturated phospholipids	[11]
japanese abricot	*Prunus mume*	Polyamines	35 °C	24 h	Pollen germination medium	Addind polyamines did not improve pollen germination	[140]

## 5. Breeding for Pollen Thermo-Tolerance

### 5.1. Heritability of Thermo-Tolerance

Since domestication started, humans tried to improve different plant traits such as disease resistance and fruit production, by selecting the most promising plants for the next generation. Nowadays, breeding for a reliable stable yield under environmental stress conditions is an important target for breeders. Global warming has increased breeder’s awareness that they should try to improve plants for tolerance to high temperatures. They do that by studying different traits such as fruit set, photosynthesis activity, and pollen quality and by looking for variation in these traits. Rudish* et al.*, 2013 [148] studied thermo-tolerance of different tomato genotypes by analysing fruit set, pollen germination and pollination. Contrasting responses to heat in the diverse genotypes made it possible to identify thermo-tolerant and thermo-sensitive genotypes. It was also shown that the tolerance can be transferred to sensitive genotypes through classical breeding. The heritability of a trait is a measure for a stable inheritance. The heritability depends on the genetic and the environmental variance in a population [149]. The heritability will be high if the genetic variance plays a relatively big role compared to the environmental variance [150]. For a breeder, a high heritability means that a trait will be stably expressed in the next generations. Several studies on the heritability of thermo-tolerance have been conducted especially in tomato. Hanson and Chen, 2002 [151] analysed the heritability of thermo-tolerance in an F2 population derived from a cross between a thermo-tolerant and a thermo-sensitive tomato cultivar. They analysed the fruit set and found a low heritability which implies that environmental conditions have a relatively big influence on thermo-tolerance compared to the genetic background of the plant. Alternatively, the low observed heritability may be due to the use of suboptimal, less robust phenotyping methods which reduces the precision of phenotyping and therefore the discriminative power to detect differences. The heritability of different parameters influencing thermo-tolerance, such as pollen viability, pollen germination and fruit set, was studied by Hazra* et al.*, 2009 [152]. They also found low heritabilities for those traits, in line with the results obtained by Hanson and Chen, 2002 [143]. Other studies, carried out with a segregating F3 population of tomato grown in two high temperature environments showed a high heritability for fruit set and fruit weight, but a low heritability for yield [145]. The authors concluded that the high heritability of those traits suggested that they can be improved by breeding. However, they also noticed that thermo-tolerance might allow fruit set, but might lead to small fruits. The challenge is to find genotypes with enhanced fruit set and with the capacity to still produce fruits with an acceptable quality. The mechanisms involved in these two processes might be different. 

### 5.2. QTL Mapping for Thermo-Tolerance

**Figure 7:**
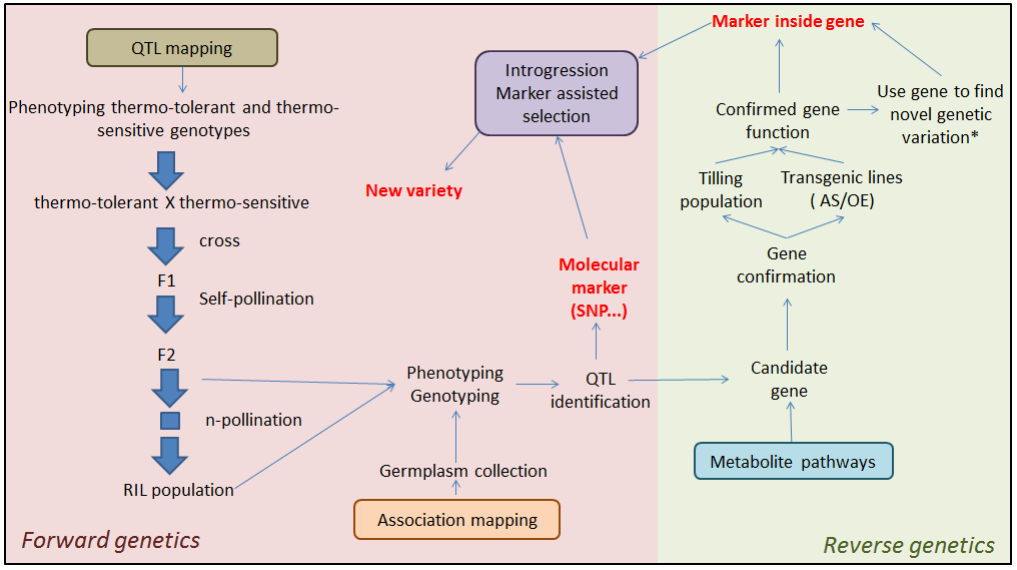
Breeding approaches to improve crop thermo-tolerance and create new thermo-tolerant varieties. QTL, quantitative trait loci; RIL, recombinant inbred lines; SNP, single-nucleotide polymorphism; AS, anti-sense; OE, over-expression. * this approach is only used in the case of reverse genetics.

Molecular markers are a useful tool for breeders to follow the introgression of specific genomic regions (Quantitative trait loci, QTL) associated with a specific trait [153]. The first step is to find QTL. This can be done by using two different forward genetics approaches (Figure 7). The first one is by crossing genotypes that are contrasting for the trait of interest and by producing mapping populations (QTL mapping). The second one is by using an available germplasm collection in which the variation of the trait of interest will be studied (association mapping). In such populations, associations can be found between molecular markers and the trait of interest. In this way, the QTL analysis can pinpoint specific chromosomal regions on which one or more genes are located that influence the trait. Subsequently, breeders can select for this trait using molecular markers, based on sequence polymorphisms in the region of interest. This is called indirect or marker-assisted selection [150]. Marker assisted selection has already been used to screen wheat varieties with markers related to grain filling duration under heat stress [154]. A marker associated with pollen thermo-tolerance could also be used to introduce thermo-tolerance into a sensitive genotype by classical breeding (Figure 7). QTL analyses for thermo-tolerance traits have been carried out (i) by measuring pollen viability, in maize [155], rice [156] and tomato [157], or (ii) by measuring fruit set, in tomato [158,159] and in rice [160,161]. QTL identification can be the starting point of more detailed analyses, for example QTL fine-mapping, which might lead to the identification of more closely-linked molecular markers which can be used by breeders to screen at the seedling stage for thermo-tolerant genotypes without too much linkage drag. Eventually, fine mapping can even lead to the isolation of the key genes underlying thermo-tolerance. In cowpea, QTLs related to thermo-tolerance have been identified by determining the number of pods per peduncle in a population of recombinant inbred lines [162]. This led to the identification of several candidate genes encoding a heat shock protein, a heat shock transcription factor and a proline transporter. The confirmation of candidate genes can be done in two different, so called, reverse genetics approaches (Figure 7). The first one is by targeting a specific gene in a tilling (targeting induced local lesions in genomes) population and analyse its phenotype. A tilling population is made by randomly mutagenizing seeds [163]. The second approach is by producing transgenic lines in which the candidate gene can be (i) silenced using for example an anti-sense construct or (ii) over-expressed using for example a constitutive or tissue-specific promoter. The phenotypic analysis of such lines may confirm that the candidate gene is indeed responsible for the variation of the trait of interest among the population. For example, if a candidate gene involved in pollen thermo-tolerance is over-expressed, a better performance under high temperature is expected compared to the wild-type. 

In addition to pollen viability or fruit set, metabolite profiles vary under different temperatures as well, particularly in pollen. The variation in levels of specific metabolites associated with thermo-tolerance can be mapped and used as an alternative way of phenotyping pollen thermo-tolerance. Firon* et al.*, 2006 [12] showed that the sugar levels are maintained in pollen of thermo-tolerant genotypes whereas in sensitive genotypes the sugar levels decreased. It was suggested that sugar level could be a good parameter for thermo-tolerance since it can be easily measured in a more accurate way than pollen viability. The identification of metabolite QTLs (mQTL) associated with thermo-tolerance could therefore be a useful tool to get novel insight into the mechanisms conferring thermo-tolerance [164,165]. In this review, several compounds that play a role in pollen thermo-tolerance, such as sugars, polyamines and hormones have been described. By studying the segregation of such metabolites in a mapping population, specific chromosomal regions can be identified which may be associated with pollen thermo-tolerance. The complex inheritance, low heritability and difficult phenotyping of thermo-tolerance make this a difficult trait to improve. The development of a good mapping population and easy and reliable phenotyping methods is the key to find thermo-tolerant QTLs and predictive molecular markers. QTL analysis is not the only way to identify candidate genes related to pollen thermo-tolerance Alternatively, detailed knowledge regarding the trait of interest may pinpoint towards specific candidate genes, whose role in pollen thermo-tolerance can be analysed through reverse genetics approaches in which the candidate gene is over-expressed or down-regulated by transgenic or tilling strategies (Figure 7). For example, in this review different metabolites have been listed for their role in pollen thermo-tolerance, such as for example flavonoids. Over-expression and/or down-regulation of genes involved in the flavonoid pathway could lead to the identification of key genes playing a role in thermo-tolerance. Such genes could form the basis to find (in diversity screens) or create (through tilling) novel genetic variation that can be used to breed for pollen thermo-tolerance, using the candidate gene as marker. 

## 6. Conclusions

Pollens are very sensitive to heat and can be affected by heat stress during different developmental stages. Thermo-tolerant and thermo-sensitive genotypes respond differently to heat stress. This is reflected in differences in metabolite accumulation profiles during development of mature fertile pollen. This review aimed to summarize the current state of the art in our understanding of the metabolic basis for pollen thermo-tolerance. To date, several metabolites have already been shown to play a role in pollen thermo-tolerance. The availability of efficient “non-targeted” metabolic screening methods make it possible to obtain a broad view on the metabolic processes involved in pollen thermo-tolerance and may lead to the identification of metabolic markers for pollen thermo-tolerance. This will allow a more efficient screening for pollen thermo-tolerance in diverse germplasm and available mapping populations and may lead to the identification of genetic markers and key genes involved in thermo-tolerance. These can be used in breeding programs aimed at improving this important trait in crop plants. 
